# Using Person-Reported Outcomes (PROs) to Motivate Young People with Diabetes

**DOI:** 10.1007/s11892-020-01305-z

**Published:** 2020-05-16

**Authors:** Maartje de Wit, Judith Versloot, Ian Zenlea, Eveline R. Goethals

**Affiliations:** 1 Medical Psychology, Amsterdam Public Health, Amsterdam UMC, Vrije Universiteit Amsterdam, Amsterdam, Netherlands; 2 Institute for Better Health, Trillium Health Partners, Mississauga, Ontario Canada; 3 Department of Paediatrics, University of Toronto, Toronto, Ontario Canada; 4 Joslin Diabetes Center, Harvard Medical School, Boston, MA USA; 5 KU Leuven, Leuven, Belgium

**Keywords:** PROs, PROMs, Person-reported outcomes, Motivation, Young people, Type 1 diabetes

## Abstract

**Purpose of Review:**

This manuscript describes how person-reported outcomes (PROs) can be utilized in care for young people with diabetes in the context of motivation.

**Recent Findings:**

The use of person-reported outcome measures (PROMS) in clinical care is feasible and acceptable, and helps focus the clinical encounter on life domains important to the person with diabetes. Results with regard to impact on self-management and glycemic outcomes are limited. Motivation is an important factor for self-management. Based on self-determination theory, autonomy-supportive, person-centered, and collaborative communication by diabetes care providers is associated with better outcomes. PROMs can facilitate this conversation.

**Summary:**

Understanding of youth motivation for maintaining or improving self-management behaviors requires a person-centered approach. PROMs can be used to facilitate an autonomy-supportive and person-centered conversation in clinical care. Training diabetes care providers in autonomy-supportive, person-centered conversation skills to discuss PROs might help to tap into youth’s motivation, but further research is needed.

## Introduction

It has well been established that type 1 diabetes (T1D) is a challenging chronic illness requiring intensive daily self-management behaviors. Maintaining or improving self-management behaviors and feeling motivated to do so represent a struggle for many. Adolescents and young adults are at high risk for self-management difficulties [1]. Analyses of registry data from the US-based T1D Exchange Study [2], European-based Diabetes-Patienten-Verlaufsdokumentation (DPV) Study in Europe [3], and a recent study in Canada [4] revealed suboptimal HbA1c values in this specific vulnerable age group. From a developmental viewpoint, adolescents and young adults living with T1D find themselves in a “*perfect storm*” of coinciding normative developmental (e.g., going to college, seeking employment, establishing romantic relationships) and illness-related challenges. Young people and their families in this transitional phase of life need to find a new balance, as diabetes management evolves from parent-driven to youth-directed diabetes care, compounded by the need for transition from pediatric to adult health care [5].

Motivation has been identified as the most common barrier to self-management [6, 7] as well as one of the most important outcomes that matter to young people with T1D [8]. Hence, to support young people with T1D to achieve optimal clinical and mental health outcomes, it is crucial for diabetes care providers to understand the components of youth motivation for behavior change as well as maintenance of healthy self-management behaviors [9–11]. This requires a person-centered care approach that emphasizes supportive communication strategies, promotes engagement, and enhances a mutual understanding among the diabetes care provider, youth, and family [11].

Person-reported outcomes (PROs) are health outcomes associated with health care or treatment that represent the person’s perspective on their health, quality of life, or functional status in a structured and standardized manner [12]. PROs are mostly referred to as patient-reported outcomes. However, in line with recent recommendations with regard to use of language in diabetes care and education [13] and to move from patient- or disease-related language to person-first language and care, we refer to PROs as person-reported outcomes [14, 15]. Over the last decade, PRO assessment has moved beyond research and has increasingly been implemented into clinical care as a means to ensure person- or family-centered care. Notably, PRO assessment with follow-up by the health care provider has been shown to positively influence well-being and satisfaction with care in young people with T1D [16–18]. Despite these recent developments in and recommendations of the use of PROs in clinical care [10, 19, 20], the literature on integration of PROs in diabetes clinical care is relatively new. Therefore, much remains to be learned about the most effective way for health care teams to utilize PROs to motivate young people with T1D and to improve self-management and glycemic outcomes [9, 21]. In this manuscript, we aim to review how PROs are currently used in care for young people with diabetes, and how they can additionally be utilized to engage young people in their care, and to motivate them for behavior change. We will illustrate our findings with an example of the implementation of a PRO assessment together with conversation skills training for diabetes care providers.

## Person-Reported Outcomes

The assessment of person-reported outcomes (PROs) provides a unique opportunity to tap into the experiences of the person with diabetes and their families, and may give the health care provider a more complete picture of the person’s unique perspective on their health and well-being [22]. The brief behavioral strategies for screening and emotional support that go hand in hand with the use of PROs may further facilitate this process [21]. Guidelines and recommendations from both pediatric and adult diabetes professional associations support assessing PROs during routine clinical care [10, 19, 20]. In 2019, the American Academy of Pediatrics (AAP) issued guidance highlighting how psychosocial factors (e.g., depression, social determinants of health) influence young people with special health care needs, and the need to assess these factors within pediatric care to limit difficulties, reduce health disparities, and promote resilience [19]. In addition, diabetes-specific guidelines from the American Diabetes Association (ADA) as well as the International Society for Pediatric and Adolescent Diabetes (ISPAD) recommend assessment of developmental progress in all domains of functioning (i.e., physical, intellectual, academic, emotional, and social development) on a routine basis [10, 20]. Reflecting the unique person perspective, PROs provide an avenue for the health care provider to directly assess and discuss these relevant psychosocial developments in a dialogue with the person and their family, instead of mainly focusing on the medical outcomes such as HbA1c and time in range [23]. Hence, PROs may broaden both the person’s and the diabetes care provider’s horizon for care and goals, and may help to move the conversation away from a problem-focused to a broader person-oriented, and strengths-based conversation. This unique person-focused approach may further enhance an individual’s feelings of empowerment, and of involvement in their treatment and goals for health behavior change. Further, including assessments of PROs in clinical diabetes care may also enhance clinician satisfaction and might even prevent provider burnout [24]. From both the person with diabetes and the clinician’s perspective, capturing PROs can improve the patient-provider relationship and facilitate shared-decision making. Additionally, implementation of measures of PROs into regular care may enhance workflow efficiency and save time. Youth completing measures before the consultation allows providers to focus on the issues that require attention during the consultation [24, 25].

### Person-Reported Outcome Measures

Person-reported outcome measures (PROMs) are standardized, validated questionnaires designed to measure PROs [26]. A distinction can be made between generic and disease-specific measures. While generic questionnaires may capture more common aspects of the person’s life and allow for comparison with normative populations, disease-specific questionnaires may be more sensitive to specific symptoms experienced by persons with diabetes [27]. In clinical practice, it depends on the purpose whether a generic and/or diabetes-specific questionnaire is most suitable.

The International Society Of Quality Of Life (ISOQOL) taxonomy of PROMs in clinical practice distinguishes different applications and types of instruments which need to be taken into account when choosing PROMs [26]. In this taxonomy, a distinction can be made between screening tools and monitoring tools. First, *screening tools* are PROMs that can help identify problems that may otherwise have gone unnoticed. Often, *preference-based measures* are used for this purpose which provide a single score, aggregated across one or multiple PRO domains, that is interpreted based on norm scores for an estimate of severity. An example is screening for depressive symptoms, where a generic depressive symptoms questionnaire with an established cut-off is used to screen young people at risk for depression [28]. PROMs used for psychosocial screening allow for care that is proactive rather than reactive, as it enables early identification of mental health symptoms and other concerns, hence, facilitating interventions and hopefully, preventing larger concerns or crises in the future [9, 29]. However, PROMs used for psychosocial screening tend to put the focus on problems, risks, and vulnerabilities and do not automatically direct the conversation to the bigger picture of living with diabetes and motivations for self-management behaviors in this context. Therefore, second to screening tools, *monitoring tools* are PROMs that can track changes over time and become an important part of person-centered care when feedback is provided not only to the clinician but also to the person with diabetes. *Profile measures* are especially suitable for this purpose as they provide multiple scores across a broad range of PRO domains [26]. An example is routine monitoring of HRQOL which provide scores across different domains that matter to the person, generic as well as diabetes-specific. This facilitates discussion between people with diabetes and clinicians regarding psychosocial concerns as well as the different domains of HRQOL in relation to diabetes self-management and well-being [17, 21, 22, 30, 31]. More recently, multi-dimensional person-centered PROMs (profile measures) are being co-developed with people living with diabetes to enable active participation and collaboration between the health care team and people with diabetes and their families [18]. The next step is implementation of PROMs in clinical care. Multiple reviews exist in the literature summarizing research regarding the use of PROMs on topics such as the use of PROs for persons and proxies in pediatric medical specialty clinics [21], the impact of PROMs on person-centered (pediatric) diabetes care [18, 22], and PROMs addressing specific topics, e.g., adherence and self-management [12]. The high-level findings of these reviews indicate that the use of PROMs is generally feasible and acceptable for young persons with diabetes, families, and diabetes care providers and helps focus the clinical encounter more on psychosocial factors and drive care decisions. However, results are mixed or limited regarding the impact on psychosocial outcomes, adherence, self-management, glycemic outcomes, and whether their use impacts referrals and access to specialty care [21, 22]. The reviews emphasize that using PROMs alone does not seem sufficient to influence clinical and psychosocial outcomes, but rather should be used to guide clinical conversations to elicit the person’s perspective about topics to inform a meaningful intervention or follow-up [18, 21].

Most PROMs, including HRQOL instruments, include items that are problem-focused, missing important positive aspects of living with diabetes (e.g., resilience, areas of strengths, self-efficacy, and feeling empowered and supported), which might facilitate conversations about motivation to change self-management behaviors. The MIND Youth-Questionnaire and the recently developed Type 1 Diabetes And Life (T1DAL) measure are some of the first PROMs to assess both positive and risk-related aspects of diabetes-related HRQOL [32, 33]. Next to these measures, PROMs taking a eudaimonic approach might tap more directly into the motivational aspects. Eudaimonic well-being focuses on appraisals of life as having meaning, purpose, and hope [34]. It has shown to be linked to autonomous motivation, psychological well-being, positive health-related behaviors, and resistance to the impact of illness [35]. Therefore, the PROMIS Pediatric Meaning and Purpose item banks could therefore be indicative of the person’s motivation as well as goal-directedness in life in general [36].

In sum, PROMs are designed and can be used for different purposes depending on the goal, such as screening or monitoring. When implemented in clinical care, a discussion of the PROMs is essential to give providers insight in aspects of the young person’s life that otherwise could have gone unnoticed and to get better insight in the motivation of young people to adhere to self-management recommendations.

### Motivation

Although few studies in diabetes research focus on the concept of motivation in youth, several valuable frameworks have been developed to conceptualize youth motivation to adhere to self-management recommendations (e.g., self-efficacy theory, theory of planned behavior) [37, 38], resulting in a wealth of concepts describing motivation (e.g., empowerment, willingness to change) [39, 40].

One such example is the self-determination theory (SDT) [41], an encompassing framework aimed at improving human motivation across multiple life domains, and particularly helpful to understand motivational processes for health behavior change [42]. SDT posits motivation to engage in certain behavior exists on a continuum from highly external (controlled) (i.e., out of a sense of pressure and to avoid negative feelings such as guilt) to highly internal (autonomous) motivation (i.e., out of a sense of personal endorsement of behaviors, and a personal understanding of their importance). Higher levels of autonomous motivation reflect a greater sense of ownership and self-endorsement of behaviors, which ultimately contributes to long-term persistence of behavior [41]. In young people with T1D, motivation and sense of ownership of diabetes management responsibilities is a constantly evolving developmental process. Therefore, it is important to be cognizant of both sides of the motivational spectrum: what drives young people to feel motivated to adhere to treatment recommendations and—on the other end of the spectrum—what drives them to *not* feel motivated or adhere. In other words, gaining insight into reasons to adhere, and their reasons not to adhere, may give clinicians valuable insight into what drives motivation in young people T1D [39]. Given this dynamic nature, systematic assessment of the person’s perspective is key and PROMs could be helpful to elicit the conversation on these topics with diabetes care providers. Important socialization figures such as parents, peers, and diabetes care providers can contribute to youth quality of motivation [43–45], and information about their role in the lives of people with diabetes may help clinicians understand the context of diabetes management. Indeed, in line with social-ecological theorizing [46, 47], young people with T1D exist within complex family and broader contextual and societal systems with multiple important stakeholders. Diabetes research is starting to focus on the impact of these stakeholders on youth motivation, as the association between youth motivation and the role of context may be theorized to be bidirectional. More specifically, derived from SDT, the communication style of diabetes guidelines by important stakeholders may play an important role [45]. For example, responsive and autonomy-supportive communication (i.e., when diabetes care providers explain the personal relevance of the diabetes-related recommendations they make to adolescents, while accepting their perspective rather than opposing possible negative feelings elicited by it [43]) is known to elicit more youth motivation. In turn, young people displaying more motivation may elicit more of such positive contextual behaviors and communication.

Research is now beginning to demonstrate that youth and parent perceptions of autonomy-supportive, person-centered, and collaborative communication, and care by diabetes care providers are associated with more optimal outcomes such as higher levels of self-efficacy and treatment adherence [45, 48, 49]. Further, there is growing insight into the power and impact of the language providers use to motivate people [13, 50]. Hence, in their clinical practice recommendation guidelines, the ADA and ISPAD now endorse person-centeredness and strengths-based communication as a fundamental component of diabetes care [10, 11, 13]. This places the person with diabetes and their family at the center, and in collaboration with diabetes care providers [11]. PROMs provide a unique opportunity to facilitate this autonomy-supportive, person-centered, and collaborative communication.

### Utilizing PROMs to Motivate Young People with Type 1 Diabetes

The use of PROMs in clinical care challenges the traditional hierarchy between the clinician and the person living with diabetes. The implementation of PROMs in clinical practice places the lived experience at the center of the clinical encounter. This translates into person-directed rather than clinician-directed goals discussed during appointments or even more broadly, goals of care. The use of PROMs facilitates the dialogue and discussion between diabetes care providers and families about these person-directed goals of care including psychosocial concerns, HRQOL, and the impact of these factors on self-management and well-being [21, 31]. Although there are no published studies examining the impact of PROMs on youth motivation, to our knowledge, using PROMs may provide a unique opportunity to tune into what young people themselves find important to focus on (or not), and, consequently, to enhance motivation for behavior change. The way in which these outcomes—“*optimal*” or “*suboptimal*”—are communicated may effectively influence a youth’s motivation for maintaining or changing diabetes-related behaviors. Following previous research on the importance of communication, we make a plea for combining the implementation of PROMs with low-threshold, and easily implementable communication training of diabetes care providers. As such, providers can be supported on how to deliver outcomes, and how to talk to young people about making behavioral changes in a way that optimally enhances their motivation to do so [13]. One way of delivering communication training could be motivational interviewing (MI) [51], a communication style consistent with an SDT inspired autonomy-supportive or person-centered communication approach for eliciting behavior change by helping people to explore and resolve ambivalence [52]. MI is aimed at initiating and maintaining partnership between the person with diabetes and the diabetes care provider, while emphasizing a caring relationship in health care communication, and by forwarding the person’s own ideas and reasons for change. Studies on MI and on person-centered communication in young people with T1D show promising results [49, 53–56], although the implementation of such provider-based interventions have proven to be difficult [21, 30, 57, 58]. PROMs might facilitate maintaining these conversation skills as their use places the person’s perspective at the center of the conversation. In order to encourage and maintain motivation, it helps to have a “strengths-based” discussion with the emphasis on successes and strengths instead of elaborating on what went wrong. It is important for clinicians to focus on how the young person can build further on these successful behaviors: What can help make things go well more often? A practical conversation guide has been proven to be helpful at the start of the implementation in clinical care to get used to the wording (see Table 1). Here, we present an example of the implementation of a diabetes-specific HRQOL PROM that is accompanied by a conversation skills training and conversation guide for clinicians, based on aspects of MI.

**Table 1:** Part of the conversation guide used in the MIND Youth-Questionnaire (MY-Q) training for clinicians

General example questions	
• I’m trying to understand, could you tell me more?	
• How does diabetes play a role in (*domain identified by youth*)	
• How do you manage? / What helped you until now?	
• When did you feel better?	
• With whom do you discuss this?	
• What are your worries about this?	
• How did you come to this score? (*referring to a specific question*)	
• What makes you rate your life with this score? What prevents you from giving a lower/higher score?	
We have now come to the end of this consultation. We dealt with several of different subject areas. You and I have concerns over (*specified domains*)	
• Would you like to receive help in this instance?	
• What would help you the most at this moment?	
◦ how can I (the team) help you with this?	

#### An Example: The MIND Youth-Questionnaire and Conversation Skills Training

Within the DAWN MIND Youth program, a manualized training for health care professionals was developed together with the MIND Youth-Questionnaire (MY-Q) [33]. The MY-Q has specifically been developed for use in clinical care and addresses as many domains of diabetes-specific HRQOL as possible with as few questions as possible. Diabetes clinics around the world have now been trained in using and implementing this PROM in their routine care [30, 59]. The training encompasses a manual that addresses the scoring and implementation of the MY-Q in clinical care as well as strategies to enhance effective communication around the MY-Q outcomes with the young person with diabetes and their family. The conversation skills training emphasizes “following” (e.g., exploring and recognizing) and “guiding” (e.g., making opening and providing alternatives) communication styles as well as strategies drawn from MI (e.g., reflective listening, supporting self-effectiveness, highlighting discrepancies). The outcomes of the MY-Q can be helpful to discuss young people’s perspectives on which domains diabetes and its treatments influence their lives. By allowing the young person to guide the conversation, the provider can discover whether there is a difference between their current behavior and future objectives (create ambivalence). Invite young people to work out their own motives, opportunities, and need for change and encourage talk of change (e.g., “I must do sports more often”). For example, aiming for perfection on all aspects of self-management is often too big a goal and an unachievable objective does not provide motivation for change, but causes frustration and discouragement. It is therefore recommended that providers limit the discussion to one or two behavioral issues at each consultation. The decision of which issues to discuss is made together with the young person. Next, the young person is responsible for choosing and implementing (the method of) change, not the provider.

A practical conversation guide helps diabetes team members in their consultation (Table 1).

Thus far, we have discussed opportunities to utilize PROMs such as HRQOL questionnaires to motivate young people with diabetes. Surprisingly few pragmatic measures have been developed to directly chart youth motivation, and their willingness (or not) to initiate or maintain healthy diabetes-related behavior. In adults with type 2 diabetes, Hessler et al. [39] validated a 9-item scale to identify individuals’ willingness to make changes, perceived ability to make or maintain changes, and feeling changes are truly worthwhile (Motivations and Attitudes Toward Changing Health scale; MATCH). Interestingly, the MATCH scale provides actionable focus points to enable a dialogue between the person with diabetes and their health care provider around behavioral change. More specifically with regard to young people, one study adapted a generic motivation questionnaire to a diabetes-related measure, but to our knowledge this measure is yet to be implemented into clinical care [43]. So more work is needed to capture the PROs on their own motivation and opportunities for change to accompany existing PROMs.

## Conclusion

Implementing PROMs in clinical care for young people with T1D is key in providing person-centered care with attention to the whole person rather than primarily focusing on glycemic control and other diabetes outcomes. The use of PROMs in clinical practice is feasible and acceptable by persons with diabetes, families, and diabetes care providers and promotes discussion about psychosocial issues and the impact of these issues on self-management. However, to our knowledge, there are no extant studies describing the relationship between the use of PROMs and youth motivation for disease management. Moreover, youth motivation could be influenced by developmental stage, and therefore, the implementation of PROMs may have differing impacts on motivation for self-management across childhood, adolescence, and young adulthood. Therefore, further studies are necessary to examine the relationship between youth motivation and PROs and whether the use of PROMs in clinical practice and at different developmental stages influences youth motivation. Finally, additional studies should examine methodologies for training important stakeholders (parents and diabetes care providers) in communication skills to address PROMs with an SDT inspired autonomy-supportive and person-centered communication approach. It is not about “the diabetes,” but about the young people and their families who live with the challenges imposed by diabetes.

## Data Availability

Not applicable.
